# Going in Blind: A Common Scenario in an Uncommon Situation

**DOI:** 10.21980/J8RS8C

**Published:** 2024-10-31

**Authors:** Ethan Hartman, Kimberly Sokol

**Affiliations:** * Kaweah Delta Medical Center, Department of Emergency Medicine, Visalia, CA

## Abstract

**Audience:**

Medical students, interns, junior resident physicians, senior resident physicians

**Background:**

Power outages have been increasing in frequency in the past few years, therefore becoming an increased threat to healthcare delivery.^1^ While most studies related to the effects of power outages are focused on outpatient care, such as acute exacerbations of chronic lung conditions and the lack of chargeable equipment, with the increasing number of power outages, hospitals must be prepared for this situation as well.^2^,^3^ Although agencies such as the Federal Emergency Management Agency (FEMA) and the US Department of Health and Human Services (HHS) have provided guidelines for the response of hospitals to temporary loss of power,^12^,^13^ hospitals generally rely on institutional policies in response to the event of a power outage. Given the relative rarity but increasing frequency of power outages in hospital settings, this medical simulation was created to present a common occurrence in the emergency department (eg, cardiac arrest) in an uncommon setting of a power outage. Simulation has been shown to improve learner self-efficacy, confidence, and leadership skills among resuscitation teams.^4^,^5^ The role of simulation also helps learners identify latent safety threats, in this case a power outage.^6^ The goal of this simulation is to improve the skills of healthcare professionals with regards to managing cardiac arrest and to encourage these practitioners to consider their own hospital guidelines in response to a power outage.

**Educational Objectives:**

By the end of this simulation, learners will be able to (1) evaluate and treat a patient experiencing myocardial infarction and subsequent cardiac arrest during a power outage, (2) describe the local protocols for managing patient care during a power outage, (3) demonstrate the ability to coordinate a medical team during a simulated power outage in an emergency department with limited resources, (4) manage a cardiac arrest patient by following Advanced Cardiac Life Support (ACLS) protocols for bradycardia and ventricular fibrillation, and (5) justify the urgency of transfer to a certified ST segment elevation myocardial infarction center/cardiac intensive care unit, referencing the recommended 120-minute door-to-balloon time.

**Educational Methods:**

This simulation was conducted with a high-fidelity mannequin. A total of six residents of various post-graduate year (PGY) levels participated in the simulated patient encounter as part of the simulation competition at the Western Regional meeting of the Society for Academic Emergency Medicine.

**Research Methods:**

This case was assessed for educational content and piloted by emergency medicine attendings from several institutions prior to running the case for the Western Regional meeting. The efficacy of the content was assessed by oral feedback.

**Results:**

The case was well-received by both the attending physicians who evaluated the case prior to running the scenario at the Western Regional meeting and the emergency medicine residents who participated in the case at the Western Regional meeting.

**Discussion:**

Overall, this simulation was well received by both the learners and the debriefers. General feedback was positive, with the perception of increased confidence among learners and reflection upon individual hospital policy in the event of a power outage.

**Topics:**

Simulation, acute myocardial infarction, cardiac arrest, power outage.

## USER GUIDE

[Table t1-jetem-9-4-s50]

**Table t1-jetem-9-4-s50:** 

List of Resources:	
Abstract	50
User Guide	52
Instructor Materials	54
Operator Materials	60
Debriefing and Evaluation Pearls	65
Simulation Assessment	67

**Learner Audience:**

Medical Students, Interns, Junior Residents, Senior Residents

**Time Required for Implementation:**

**Instructor Preparation:** 15 minutes

**Time for case:** 15 minutes

**Time for debriefing:** 15 minutes

**Recommended Number of Learners per Instructor:** up to 6:1

**Topics:**

Simulation, acute myocardial infarction, cardiac arrest, power outage.

**Objectives:**

By the end of this simulation session, learners will be able to:

1. Evaluate and treat a patient experiencing myocardial infarction and subsequent cardiac arrest during a power outage.
2. Describe the local protocols for managing patient care during a power outage.
3. Demonstrate the ability to coordinate a medical team during a simulated power outage in an emergency department with limited resources.
4. Manage a cardiac arrest patient by following ACLS protocols for bradycardia and ventricular fibrillation.
5. Justify the urgency of transfer to a certified ST segment elevation myocardial infarction center/cardiac intensive care unit, referencing the recommended 120-minute door-to-balloon time.

### Linked objectives and methods

During this simulation, the participants will need to manage a patient with a myocardial infarction and subsequent cardiac arrest during a power outage (objective 1). Seeing as how the power outage and cardiac arrest will occur simultaneously, team members will likely be surprised or shocked, forcing the learners to calm the room and effectively lead their team through the chaos (objectives 2 and 3). They will then need to use their ACLS algorithms to attain return of spontaneous circulation (objective 4), ultimately ensuring that the patient can get to a STEMI center within 120 minutes of their heart attack (objective 5).

### Recommended pre-reading for instructor

The instructor should read ACLS algorithms and understand differing hospital policy guidelines regarding power outages and natural disasters.^7^–^9^ If this is being conducted at a single institution, the instructor should be aware of that institution’s guidelines for power outages (if present).

### Results and tips for successful implementation

This simulation is best implemented in an educational environment that includes high-fidelity simulation resources with monitors that will allow the learners to interpret vital signs. Participants in the scenario should be in groups of four to six, and they are expected to self-assign roles throughout the scenario to best manage the team. In order to simulate a power outage without sacrificing safety of the participants or simulation staff, we recommend turning off as many lights as possible with a central switch while still having some emergency lighting on in the background. If possible, consider also having exogenous electronics on at the start of the scenario (i.e., electric fan, music from a radio) so that when they are suddenly turned off, the effect will be even more dramatic. We also suggest including sounds of inclement weather, such as rain or wind to provide context for the power outage.

The simulation scenario was initially piloted by a group of emergency medicine attendings, then participated in by a group of emergency medicine residents, in their first-through-fourth years of training, in front of a live audience of emergency residents and attending physicians and an expert panel of judges. Debrief was provided by the narrator and the judges with specific learning points. Immediately after the simulation, we obtained oral feedback from the participants, judges, and audience members to assess content. The overall feedback on the simulation was positive and well-received.

## Figures and Tables

**Table t2-jetem-9-4-s50:** 

#1	EKG of inferior STEMI with third degree heart block (reference noted with stimulus)

**Table t3-jetem-9-4-s50:** 

**Vitals:** Heart Rate (HR) 52	Blood Pressure (BP) 90/60
Respiratory Rate (RR) 22	Temperature (T) 98.6°F
Oxygen Saturation (O2Sat) 94%	

**Figure f1-jetem-9-4-s50:**
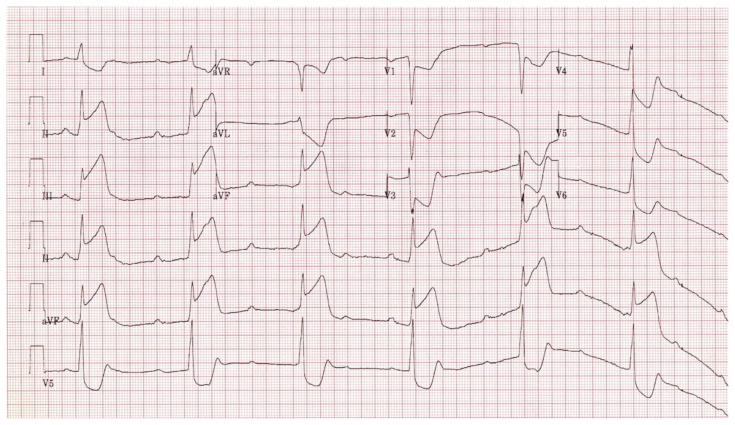


**Table t4-jetem-9-4-s50:** SIMULATION EVENTS TABLE:

Minute (state)	Participant action/ trigger	Patient status (simulator response) & operator prompts	Monitor display (vital signs)
0:00 (Baseline)	Patient is sitting on the gurney in the ED. Participants should assess the patient’s airway, breathing, and circulation while asking the nurse to place the patient on a monitor and obtain vitals. Participants should perform a focused history and physical exam.	Can play thunderstorm sounds on phone/speakers to simulate the storm, and add to the chaos: https://www.youtube.com/watch?v=Np7iHS0wyzU Nurse should continue to comment on the storm outside, with statements such as, “Wow, it is really coming down outside,” or even, “I hope the power doesn’t go out again - it tends to do that!” If participants do not ask, the nurse should prompt them to place the patient on a monitor and obtain vitals.	T 98.6°F HR 52 (lead II, with inferior STEMI, 1^st^ degree heart block on monitor) BP 90/60 RR 22 O2 94%
1:00 (State 1)	Change to 2^nd^ degree block for 30 seconds, then to 3^rd^ degree block for 30 seconds. Participants order CXR, EKG, and labs, including CBC, CMP, and troponin. Participants ask nurse to place a peripheral IV.	Potential nursing prompts if the participant does not complete critical action: If no labs ordered: “Doctor, would you like to order anything? If no EKG is ordered: “Would you like to get an EKG?” If no CXR ordered: “Is there any imaging you want?” If cardiac workup labs are not ordered: “Are there any tests you would like?” “Doctor, do you think this could be a cardiac issue? I just saw a patient like this who was having a heart attack.” If participants don’t notice the rhythm change, nurse can say, “Hey doc, can you look at the rhythm? It doesn’t look so good!” If participants attempt to give nitro, the nurse can say “Doc, I’m uncomfortable with that - it appears that the patient’s blood pressure is too low to give nitro.”	T 98.6°F HR 52 (lead II, with inferior STEMI, 2^nd^ → 3^rd^ degree) BP 90/60 RR 22 O2 94%
2:00 (State 2)	Participants should place patient on supplemental oxygen and manage patient’s bradycardia and hypotension with atropine and intravenous fluids.	If participants don’t recognize that the patient is clinically deteriorating, the nurse should prompt: “His blood pressure appears to be dropping,” or, “his heart rate appears to be slowing down.”	T 98.6°F HR 43 (lead II, with inferior STEMI, 3^rd^ degree heart block) BP 90/60 RR 22 O2 80% on RA (can increase to 89% if placed on oxygen)
3:00 (State 3)	Power will turn off during this stage. Participants initiate CPR/ACLS protocol once patient loses pulses. Participants place patient on a portable monitor, or ensure their current monitor is plugged in a red outlet, which should theoretically still be working in a power outage. Participants perform endotracheal intubation with either direct or video laryngoscopy, ensuring that the scope is plugged in to a red outlet.	Patient states, “I don’t feel so good” and then becomes unresponsive, going into ventricular fibrillation cardiac arrest. At this point, the monitor briefly shows ventricular fibrillation prior to turning off, along with the power in the rest of the emergency department. If the participants attempt to use a video laryngoscope without plugging it in to a red outlet (or vocalizing this step), the nurse needs to apologize, stating that the machine hadn’t been plugged in prior to the power outage and is therefore not fully charged. Consider background noises (eg, fans, music, beeping etc.), so when power goes out, the scenario is notably much quieter.	T 98.6°F HR 0, ventricular fibrillation on monitor BP 0 RR 0 (or rate of bagging once intubated) Maximum O2 sat will be 80% with BVM breaths and 90% after endotracheal intubation
4:00–10:00 (State 4)	Participants continue ACLS protocol for ventricular fibrillation arrest, with defibrillation, administration of epinephrine and amiodarone, and rhythm checks every 2 minutes for a total of 3 rounds.	If participants do not follow ACLS protocol correctly, the nurse can prompt them to use a memory tool such as a phone application or a card on which is written ACLS protocols. If the participants stop conducting ACLS protocol (i.e., if distracted by the power outage), the nurse should instruct them to continue.	T 98.6°F HR 0, ventricular fibrillation on monitor BP 0 RR 0 (or rate of bagging once intubated) Maximum O2 sat will be 80% with bag valve mask (BVM) breaths and 90% after endotracheal intubation
10:00 (Case Completion)	Participants should vocalize the importance of transferring the patient to a STEMI center and ask for help in facilitating this.	Power returns. Patient regains pulses; however, still with a STEMI. If the participants do not start to talk about post-ROSC care, the nurse can prompt them with: “What’s the plan for this patient?”	T 98.6°F HR 72 (lead II, with inferior STEMI, 3^rd^ degree heart block on monitor) BP 110/80 RR 20 O2 94%

**Table t5-jetem-9-4-s50:** 

**Critical Actions:** Assess patient’s airway, breathing, and circulation. Place the patient on supplemental O2. Place the patient on a cardiac monitor. Perform a focused history and physical examination. Order EKG, blood work, chest x-ray (CXR) while initiating treatment. Correctly manage ventricular fibrillation: Start chest compressions within 10 seconds of loss of pulses. Place patient on defibrillator pads and the portable defibrillator monitor, including portable O2 saturation. Manage patient’s airway with definitive airway (perform endotracheal intubation). Minimize interruptions in chest compressions. Perform defibrillation when indicated. Given amiodarone and epinephrine when indicated. Appropriately manage the scene during power outage. Move patient from monitoring system to portable monitor OR plug monitor into a back-up source system (i.e., red outlet). Ensure safety of teammates throughout power outage. Initiate post-ROSC management, including a discussion regarding thrombolytics (ultimately no need as the patient can arrive at the nearest STEMI center within 90 minutes), targeted temperature management, initiating vasopressors for blood pressure control, and transfer to a higher level of care.	0:00

**Table t6-jetem-9-4-s50:** 

	Milestone	Did not achieve level 1	Level 1	Level 2	Level 3
1	**Emergency Stabilization (PC1)**	□ Did not achieve Level 1	□ Recognizes abnormal vital signs	□ Recognizes an unstable patient, requiring intervention Performs primary assessment Discerns data to formulate a diagnostic impression/plan	□ Manages and prioritizes critical actions in a critically ill patient Reassesses after implementing a stabilizing intervention
2	**Performance of focused history and physical (PC2)**	□ Did not achieve Level 1	□ Performs a reliable, comprehensive history and physical exam	□ Performs and communicates a focused history and physical exam based on chief complaint and urgent issues	□ Prioritizes essential components of history and physical exam given dynamic circumstances
3	**Diagnostic studies (PC3)**	□ Did not achieve Level 1	□ Determines the necessity of diagnostic studies	□ Orders appropriate diagnostic studies. Performs appropriate bedside diagnostic studies/procedures	□ Prioritizes essential testing Interprets results of diagnostic studies Reviews risks, benefits, contraindications, and alternatives to a diagnostic study or procedure
4	**Diagnosis (PC4)**	□ Did not achieve Level 1	□ Considers a list of potential diagnoses	□ Considers an appropriate list of potential diagnosis May or may not make correct diagnosis	□ Makes the appropriate diagnosis Considers other potential diagnoses, avoiding premature closure
5	**Pharmacotherapy (PC5)**	□ Did not achieve Level 1	□ Asks patient for drug allergies	□ Selects an medication for therapeutic intervention, consider potential adverse effects	□ Selects the most appropriate medication and understands mechanism of action, effect, and potential side effects Considers and recognizes drug-drug interactions
6	**Observation and reassessment (PC6)**	□ Did not achieve Level 1	□ Reevaluates patient at least one time during case	□ Reevaluates patient after most therapeutic interventions	□ Consistently evaluates the effectiveness of therapies at appropriate intervals
7	**Disposition (PC7)**	□ Did not achieve Level 1	□ Appropriately selects whether to admit or discharge the patient	□ Appropriately selects whether to admit or discharge Involves the expertise of some of the appropriate specialists	□ Educates the patient appropriately about their disposition Assigns patient to an appropriate level of care (ICU/Tele/Floor) Involves expertise of all appropriate specialists
9	**General Approach to Procedures (PC9)**	□ Did not achieve Level 1	□ Identifies pertinent anatomy and physiology for a procedure Uses appropriate Universal Precautions	□ Obtains informed consent Knows indications, contraindications, anatomic landmarks, equipment, anesthetic and procedural technique, and potential complications for common ED procedures	□ Determines a back-up strategy if initial attempts are unsuccessful Correctly interprets results of diagnostic procedure
20	**Professional Values (PROF1)**	□ Did not achieve Level 1	□ Demonstrates caring, honest behavior	□ Exhibits compassion, respect, sensitivity and responsiveness	□ Develops alternative care plans when patients’ personal beliefs and decisions preclude standard care
22	**Patient centered communication (ICS1)**	□ Did not achieve level 1	□ Establishes rapport and demonstrates empathy to patient (and family) Listens effectively	□ Elicits patient’s reason for seeking health care	□ Manages patient expectations in a manner that minimizes potential for stress, conflict, and misunderstanding. Effectively communicates with vulnerable populations, (at risk patients and families)
23	**Team management (ICS2)**	□ Did not achieve level 1	□ Recognizes other members of the patient care team during case (nurse, techs)	□ Communicates pertinent information to other healthcare colleagues	□ Communicates a clear, succinct, and appropriate handoff with specialists and other colleagues Communicates effectively with ancillary staff

## INSTRUCTOR MATERIALS

**Case Title:** Going in Blind: A Common Scenario in an Uncommon Situation

**Case Description & Diagnosis (short synopsis):** During a severe thunderstorm, 60 minutes away from the nearest STEMI center, a 58-year-old male with a past medical history of coronary artery disease and diabetes mellitus presents to a critical access facility with chest pain and is quickly found to have an acute inferior STEMI. The patient will arrive in first degree heart block, then continue to decompensate soon after arrival to the ED, first going into Wenckebach, then third degree heart block, and ultimately going into cardiac arrest. At around the same time the patient codes, the lights and monitors in the emergency room go out due to a massive power outage. Despite the lack of power and using the limited resources available to them, the team should continue to code the patient according to ACLS protocol. At the ten-minute mark, the patient will achieve return of spontaneous circulation (ROSC), and the power will return, thus ending the case.

**Equipment or Props Needed:**

- High-fidelity simulation mannequin
- Power strip with a switch connected to most devices
- Monitor
- Manual Blood Pressure cuff
- Stethoscope
- Portable SPO2 monitor
- Portable DL blade with light
- Intubation materials (including suction and 7.5 cuffed endotracheal tube (ETT)
- Crash cart complete with portable monitor/defibrillator, medicines (eg, epinephrine, amiodarone, lidocaine, etc.), and intubation materials

**Extra considerations:** Water on the patient’s forehead to demonstrate diaphoresis. Consider other electrical devices such as a loud fan, television, and/or radio with music playing to further dramatize the power outage.

**Embedded participants needed:**

**Nurse:** Responds only if using closed-loop communication. Will speak at certain times if not prompted specifically. Will prompt the provider with certain critical action item questions should the provider not ask directly. Will start IVs, draw labs, activate code, alter compressions as appropriate.

**Stimulus Inventory:** [Table t2-jetem-9-4-s50]

**Background and brief information:** The scenario takes place in the emergency department of a small, critical access facility with minimal staffing. The patient is a 50-year-old male who arrives via private auto with a chief complaint of crushing chest pain.

**Initial presentation:** The patient initially presents with chest pain for two hours that is described as crushing. He has radiation of pain to the left arm. He notes difficulty breathing, sweating, and nausea, and his physical exam is consistent with an uncomfortable middle-aged man who is diaphoretic and bradycardic.

**How the scene unfolds:** The patient arrives to the ED in first degree heart block. Participants should get an EKG soon after arrival to confirm the first-degree heart block as well as recognize the patient is suffering from a STEMI. They should treat the patient with aspirin and avoid nitroglycerin given the patient’s presenting hypotension. Regardless, the patient will continue to decompensate soon after arrival to the ED, first going into Wenckebach, then 3rd degree heart block, and ultimately going into cardiac arrest despite correct treatment by the participants. At around the same time, the patient codes, the lights and monitors in the emergency room go out due to a massive power outage. Using the limited resources available to them, the team should continue to code the patient according to ACLS protocol despite the lack of power. If the team correctly follows the ACLS protocol for ventricular fibrillation, then the patient may achieve ROSC at 10 minutes time, prompting the team to arrange for transfer to a tertiary care center for definitive STEMI management.

**Anticipated management mistakes:**

- Difficulty with bedside monitors/portable monitoring: Participants may have difficulty locating/placing the patient on the Zoll monitor, portable SPO2 monitor, and/or portable blood pressure cuff.
- Difficulty with visualization of necessary equipment or vocal cords during intubation: May require assistance.
- Failure to recognize the clinical status change to pulselessness and ventricular fibrillation: Participants may require prompting to call a code and start CPR per ACLS.
- Inappropriate medication or defibrillation: Participants may make incorrect choices in medication or fail to defibrillate appropriately, in which case the patient will not achieve ROSC.

**Critical actions:**

1. Assess patient’s airway, breathing, and circulation.
2. Place the patient on supplemental O2.
3. Place the patient on a cardiac monitor.
4. Perform a focused history and physical examination.
5. Order EKG, blood work, chest x-ray (CXR) while initiating treatment.
6. Correctly manage ventricular fibrillation:
  - Start chest compressions within 10 seconds of loss of pulses.
  - Place patient on defibrillator pads and the portable defibrillator monitor, including portable O2 saturation.
  - Manage patient’s airway with definitive airway (perform endotracheal intubation).
  - Minimize interruptions in chest compressions.
  - Perform defibrillation when indicated.
  - Given amiodarone and epinephrine when indicated.
7. Appropriately manage the scene during power outage.
  - Move patient from monitoring system to portable monitor OR plug monitor into a back-up source system (i.e., red outlet).
  - Ensure safety of teammates throughout power outage.
  - Initiate post-ROSC management, including a discussion regarding thrombolytics (ultimately no need as the patient can arrive at the nearest STEMI center within 90 minutes), targeted temperature management, initiating vasopressors for blood pressure control, and transfer to a higher level of care.

**Case Title:** Going in Blind: A Common Scenario in an Uncommon Situation

**Chief Complaint:** Chest Pain[Table t3-jetem-9-4-s50]

**General Appearance:** Uncomfortable, diaphoretic

**Primary Survey:**

- **Airway:** Intact
- **Breathing:** Symmetrical chest rise, equal breath sounds bilaterally
- **Circulation:** Distal pulses weak and thready in all extremities

**History:**

- **History of present illness:** 50-year-old male with a history of hypertension (HTN), hyperlipedemia (HLD), diabetes mellitus (DM), and coronary artery disease (CAD) status post several stents initially presents with chest pain for two hours that began at rest and is described as crushing. He has radiation of pain to the left arm. He notes difficulty breathing, sweating, and nausea.
- **Past medical history:** HTN, HLD, DM, CAD
- **Past surgical history:** Cholecystectomy, Cardiac stent placed x 2, two years ago, patient unsure of which vessels or doctor
- **Patient’s medications:** Atorvastatin, Hydrochlorothiazide (HCTZ), Metformin, Aspirin, Nitroglycerin as needed for chest pain
- **Allergies:** None
- **Social history:** Smoke cigarettes one pack per day, drinks three beers per day, more on weekends, Works in construction
- **Family history:** Father died of a heart attack at age 55; Mother has diabetes

**Secondary Survey/Physical Examination:**

- **General Appearance**: Middle-aged male in moderate acute distress, actively working to breathe, diaphoretic
- **HEENT**
  ○ Head: within normal limits
  ○ Eyes: within normal limits
  ○ Ears: within normal limits
  ○ Nose: within normal limits
  ○ Throat/oropharynx: within normal limits
- **Neck**: within normal limits
- **Heart**: Bradycardic rate, pulses are weak and thready but symmetric
- **Lungs**: Increased work of breathing and tachypnea due to pain only, otherwise normal
- **Abdominal/GI**: within normal limits
- **Genitourinary**: within normal limits
- **Rectal**: within normal limits
- **Extremities**: Slightly mottled with a decreased capillary refill, otherwise normal
- **Neuro**: within normal limits
- **Skin**: Diaphoretic, otherwise normal
- **Lymph**: within normal limits
- **Psych**: within normal limits

Note: While the participants should order labs and additional studies, only the EKG as shown below will result during the short duration of the case.

*Electrocardiogram*

Larkin J and Buttner R. AV Block: 3^rd^ degree (complete heart block). In: Life in the Fast Lane. Published November 15, 2023. Accessed August 22, 2024. CC BY-NC-SA 4.0. At: https://litfl.com/av-block-3rd-degree-complete-heart-block/.[Fig f1-jetem-9-4-s50]

## OPERATOR MATERIALS

[Table t4-jetem-9-4-s50]

**Diagnosis:**

Cardiac arrest secondary to acute myocardial ischemia

**Disposition:**

Transfer to higher level of care

## DEBRIEFING AND EVALUATION PEARLS

*Going in Blind: A Common Scenario in an Uncommon Situation*

**Pearls:**

**STEMI Pearls:**

- - ST-elevation myocardial infarction = occlusion of one or more of the coronary arteries that supply the heart with blood, likely through plaque rupture or dissection of the coronary arteries leading to an obstructing thrombus^10^
- - Diagnostic criteria for STEMI^10^:
  ○ New ST-segment elevation at the J point in 2 contiguous leads with specific cutoff points based on age and gender
  ○ For patients with a pre-existing left bundle branch block, Sgarbossa’s criteria is used
- - Initial management:
  ○ If hypoxic – place on oxygen (but avoid in normoxic patients)^10^
  ○ Percutaneous coronary intervention (PCI):
    ▪ Goal of 90 minutes of initial patient presentation at a PCI capable hospital^10^
    ▪ Goal of 120 minutes of initial presentation if transfer is required^10^
    ▪ If PCI is not possible within these time frames, consider fibrinolytic therapy within 30 minutes of patient arrival^10^

**ACLS Pearls:**

- - Bradycardia – ensure you are identifying and treating the underlying cause of the bradycardia, while managing symptomatic patients with atropine (1mg every 3–5 minutes with a max of 3mg), transcutaneous/transvenous pacing, and/or dopamine or epinephrine infusions^7^
- - Ventricular fibrillation – perform high quality CPR, ensuring quick rhythm assessment and defibrillation, with administration of epinephrine (1mg every 3–5 minutes) and amiodarone (first dose 300mg, second dose 150mg), and obtaining an advanced airway while investigating for reversible causes^7^

**Power Outage Pearls:**

- - Use the five elements of crew resource management (communication, specifically closed-loop communication, situational awareness, decision making, teamwork, addressing barriers) when serving as a leader in a chaotic environment such as a power outage^11^
- - Communication tips:
  ○ Establish communication in a centralized location such as an incident command center
  ○ Designate a leader within the hospital not directly involved with patient care that is solely responsible for ensuring the effective distribution of information
  ○ Ensure downtime communication protocols are in effect as computers will likely be down – this includes writing key information about each patient such as abnormal vitals in their rooms, while still ensuring patient privacy
- - Secondary power sources:
  ○ Pool all light sources so that hospital personnel have easy access to lighting to ensure safety of the team – this includes smart phones, pen lights, flashlights, portable lights typically used for pelvic exams
  ○ Red outlets are typically used to indicate those connected to the emergency power supplies – ensure all key equipment is plugged into those outlets
  ○ If in doubt about secondary power sources, contact your hospital’s facility management and/or clinical engineering department, who are likely the lead in electrical-related issues
- - Factors causing the power outage:
  ○ Inclement weather – ensure the safety of emergency medical services workers, hospital employees, and patients, especially those requiring transfer to other facilities
  ○ Anticipate the needs of your community – there may be increased patients due to ventilator-dependent residents at nursing homes, inclement weather-related incidents, etc.
- - Instructor tip: Consider incorporating your own institution’s contingency plan for power outages into the debrief, if present; if not, consider coming up with your own action plan, using the templates provided by the California Hospitals Association and OSHA as a guide^7^,^8^

## SIMULATION ASSESSMENT

*Going in Blind: A Common Scenario in an Uncommon Situation*

Learner: _________________________________________

***Assessment Timeline***

This timeline is to help observers assess their learners. It allows observer to make notes on when learners performed various tasks, which can help guide debriefing discussion.[Table t5-jetem-9-4-s50]

**Critical Actions:**

□ Assess patient’s airway, breathing, and circulation.
□ Place the patient on supplemental O2.
□ Place the patient on a cardiac monitor.
□ Perform a focused history and physical examination.
□ Order EKG, blood work, chest x-ray (CXR) while initiating treatment.
□ Correctly manage ventricular fibrillation:
  □ Start chest compressions within 10 seconds of loss of pulses.
  □ Place patient on defibrillator pads and the portable defibrillator monitor, including portable O2 saturation.
  □ Manage patient’s airway with definitive airway (perform endotracheal intubation).
  □ Minimize interruptions in chest compressions.
  □ Perform defibrillation when indicated.
  □ Give amiodarone and epinephrine when indicated.
□ Appropriately manage the scene during power outage.
□ Move patient from monitoring system to portable monitor OR plug monitor into a back-up source system (i.e., red outlet).
□ Ensure safety of teammates throughout power outage.
  □ Initiate post-ROSC management, including a discussion regarding thrombolytics (ultimately no need as the patient can arrive at the nearest STEMI center within 120 minutes), targeted temperature management, initiating vasopressors for blood pressure control, and transfer to a higher level of care.

**Summative and formative comments:**

**Milestones assessment:** [Table t6-jetem-9-4-s50]
